# Associations between Parent-Adolescent Attachment Relationship Quality, Negative Life Events and Mental Health

**DOI:** 10.1371/journal.pone.0080812

**Published:** 2013-11-29

**Authors:** Rienke Bannink, Suzanne Broeren, Petra M. van de Looij – Jansen, Hein Raat

**Affiliations:** 1 Department of Public Health, Erasmus University Medical Center Rotterdam, Rotterdam, the Netherlands; 2 Municipal Public Health Service Rotterdam area, Rotterdam, the Netherlands; The University of Queensland, Australia

## Abstract

**Purpose:**

The aim of this study was to examine the association of negative life events and parent-adolescent attachment relationship quality with mental health problems and to explore an interaction between the parent-adolescent attachment relationship and one or multiple negative life events on the mental health of adolescents.

**Methods:**

A two-year longitudinal study was conducted among first-year secondary school students (N = 3181). The occurrence of life events and the quality of parent-adolescent attachment were assessed at baseline and mental health status at two-year follow-up by means of self-report questionnaires. Binary logistic regression analyses were conducted to assess associations between life events, parent-adolescent attachment and mental health problems. Relative Excess Risk due to Interaction techniques were used to determine the interaction effects on the additive scale.

**Results:**

Life events were related to mental health status, as was parent-adolescent attachment. The combined effect of an unfavourable parent-adolescent attachment with life events on mental health was larger than the sum of the two individual effects. Among adolescents with one life event or multiple life events, an unfavourable parent-adolescent attachment increased the risk of mental health problems at follow-up compared to the group without life events.

**Conclusion:**

Results supported an interaction effect between parent-adolescent attachment and negative life events on mental health. Especially adolescents with one or multiple life events and an unfavourable parent-adolescent attachment seems to be a vulnerable group for mental health problems. Implications for further research are discussed.

## Introduction

An estimated 15% of adolescents in the Netherlands have mental health problems [1]. Mental health problems often have their first manifestation during adolescence [2] and are associated with serious co-morbidity including underachievement in age-appropriate social skills, delinquency and an elevated risk of suicide [3], . Mental health problems in adolescence pose a risk for the development of psychiatric disorders in adulthood [5]–[8]. Understanding determinants of mental health problems, such as risk and protective factors, is important for the prevention of these problems.

One important risk factor for psychopathology that has been under investigation for many years is the impact of negative life events [9], [10]. Results from several studies indicate an association in adolescents between negative life events and mental health problems. Examples of such life events that have been posited as risk factors for developing mental health problems in previous studies are physical illness of a parent [11], parental psychiatric illness [12], [13], parental substance use [14], [15], family breakdown [16], parental conflicts [17], [18] and early parenthood [19].

Longitudinal studies have also identified factors positively influencing the mental health of adolescents [20]–[23]. One of these factors is parent-adolescent attachment relationship quality. Previous research has shown that a favourable parent-adolescent attachment relationship may serve as a protective factor for mental health problems [21], [22], [24], [25], with the quality of adolescents’ attachment with parents having an impact on their current mental health status, as well as on their prospect of developing mental health problems, such as major depression, later in life [23].

Although there is a considerable amount of literature examining the simple association of life events and parent-adolescent attachment with mental health problems in isolation, most studies fail to examine the interaction between protective and risk factors such as parent-adolescent attachment and life events on mental health. Instead of concentrating on risks factors in isolation, increasing research attention is devoted to factors that promote health and their interaction with risk factors. This corresponds to research on resilience within the field of developmental psychology. Research on resilience focuses on adolescents who show positive developmental outcomes despite experiencing significant adversity [26], [27].

Social support, for example, is seen as one of these resilience factors and is theorized to protect adolescents from the impacts of stress. Support from parents is thought to operate by lessening the threat children experience when encountering stress, thereby leading to more adaptive coping efforts [28]. Finally, families who provide adequate support meet adolescent’s needs for safety and security and may empower adolescents by bolstering their sense of self-esteem or control [29].

The parent-adolescent attachment relationship is another potential resilience factor that warrants further research attention. It is essential to understand the role that parent-adolescent attachment plays in the relationship between life events and mental health, because while life events often cannot be avoided, parent-adolescent attachment is amendable [30]. If a favourable parent-adolescent attachment could buffer the association between life events and mental health problems, this could help to distinguish vulnerable adolescents from those with good adaptation under extenuating circumstances, and could have implications for preventing and treating mental health problems in adolescents.

One initial study has examined the buffering role of multiple protective factors, including parental support, on mental health among adolescents with or without life events [31]. Wille et al. [31] compared the percentages of mental health problems in adolescents with different numbers of life events while taking into account the availability of protective factors. Protective factors were found to significantly buffer the association between adolescents exposed to one or two life events and mental health. Adolescents without a life event did not benefit from the availability of protective factors. The current study capitalizes on a large study and differs from Wille et al. by specifically quantifying the additional effect that the interaction between parent-child attachment and life events may have on mental health. In line with previous findings, we hypothesize that a favourable parent-adolescent attachment (i.e. the protective factor) will buffer the association between life events and mental health. The goals of this study were 1) to examine the association of negative life events and parent-adolescent attachment relationship quality with mental health problems and 2) to investigate if there is an interaction between the parent-adolescent attachment relationship and one or multiple negative life events on the mental health of adolescents.

## Methods

### Design and participants

A prospective study with a two-year follow-up was conducted as part of the Rotterdam Youth Monitor (RYM), a longitudinal youth health surveillance system. The RYM monitors the general health, well-being, behaviour and related factors of youth aged 0 to 19 years living in Rotterdam and the surrounding region in the Netherlands. The RYM is incorporated into the preventive care (regular health examinations) of the preventive youth healthcare system; the RYM is used to detect potential individual health risks and problems in order to take necessary preventive measures (including referrals for treatment).

The current study used RYM data from students at secondary schools. At baseline, the students were in the first year of secondary education (*M*_age_ = 12.5 years, *SD* = 0.62), and at follow-up (Year 2) in the third year (*M*_age_ = 14.3 years, *SD* = 0.58). Data were collected throughout the school year, except in the months of July and August (Dutch summer holidays). The students completed a baseline questionnaire between September 2008 and July 2009 and a follow-up questionnaire between September 2010 and July 2011. Administration of the questionnaire at schools was conducted by specially trained researchers and school nurses from the Municipal Public Health Service and/or by a teacher. In 2008−2009, 8,272 adolescents participated (95% participation rate), of whom 3,181 participated again in 2010−2011 (38%). The main reason for non-response at baseline was students’ illness at the time of administering the questionnaire. The main reason for non-response at follow-up was that schools were not willing to participate at follow-up. Other reasons were: students had transferred to a school that did not participate at follow-up, students had repeated a school year or students were absent at the time of administering the follow-up questionnaire.

### Ethics statement

All data were gathered within and as part of the government approved routine health examinations of preventive youth health care; the RYM was completed on a voluntary basis; anonymous data were used in this study; separate informed consent was therefore not requested. Adolescents received verbal information on the RYM, each time it was applied; their parents received written information on the RYM, each time it was applied; both adolescents and their parents were free to object to participation.

### Measures

**Mental health problems.** Mental health was assessed at follow-up by the Dutch self-report version of the Strengths and Difficulties Questionnaire (SDQ) [32]–[36]. The SDQ consists of 25 items for describing positive and negative attributes of adolescents that can be allocated to five subscales of five items each. The subscales are: emotional problems, conduct problems, hyperactivity-inattention, peer problems, and prosocial behaviour. Each item has to be scored on a three-point scale, with 0 =  ‘*not true*’, 1 =  ‘*somewhat true*’, and 2 =  ‘*certainly true*’. A total difficulties score can be calculated by adding up the scores on the emotional problems, conduct problems, hyperactivity-inattention and peer problems subscales (range 0−40; current study *α* = 0.74).

Two groups were created based on the total SDQ score: normal (cut-off point SDQ at follow-up ≤ 80^th^ percentile; score ≤ 13) and borderline/abnormal mental health problems (cut-off point SDQ at follow-up > 80^th^ percentile; score ≥ 14) [37]. Cut-off points were based on a previous large cross-national survey among 14−15 year old adolescents [1].

**Life events.** Adolescents were asked about 11 negative life events, which were measured using three different types of response categories. Each life event was assessed at baseline with one item. For six of the life events (i.e. chronic or severe illness of parent, chronic or severe illness of sibling, mental illness of parent, mental illness of sibling, addiction to alcohol, drugs and/or gambling of parent, addiction to alcohol, drugs and/or gambling of sibling), the possible responses were: *not true*, *not currently true* and *true*. For analysis, these items were dichotomized into: not (currently) true, and true. For two life events (i.e. regular conflicts between parents, parental divorce), possible responses were: *not experienced*, *experienced > 2 years ago* and *experienced ≤ 2 years ago*. For analysis, regular conflicts between parents was dichotomized into: not experienced or > 2 years ago, and experienced ≤ 2 years ago. Parental divorce, as well as three other life events (i.e. unwanted pregnancy, victim of sexual abuse and victim of violence), were categorized as: no and yes.

A total life event score was calculated by adding up the dichotomized item scores. Subsequently, three groups were created based on the total life event scores: no life event, one life event or multiple life events.

**Parent-adolescent attachment relationship.** Parent-adolescent attachment relationship quality was measured at baseline using the ‘Family attachment scale’ of The Communities That Care Youth Survey [38], [39]. This scale consists of six items: three items about the adolescent’s relationship with the mother and three items about the relationship with the father. The items were scored on the four-point scale using: *NO!*, *no*, *yes*, *YES!*. A total score could be calculated by taking the average of the six items (range 0 – 3; current study *α* = 0.82). This scale was dichotomized based on the sample distribution in this study: unfavourable parent-adolescent attachment (cut-off point < 20^th^ percentile; score < 2.00) and favourable parent-adolescent attachment (cut-off point ≥ 20^th^ percentile; score ≥ 2.00).

**Confounders.** Age, gender, ethnicity, and education level of the adolescent were measured at baseline and are incorporated in this study as potential confounders. For analysis purposes, confounders were dichotomized. Age was dichotomized into the categories below 13 years and 13 years or older. Education was categorized into two groups: basic or theoretical pre-vocational education, and general secondary/pre-university education [40]. Ethnicity was classified as Dutch or non-Dutch. In accordance with the definitions of Statistics Netherlands [41], adolescents with at least one parent born outside the Netherlands were classified as non-Dutch.

### Statistical analysis

Descriptive statistics were calculated for general characteristics of the study population (Table 1). Differences in gender, age, ethnicity, education, life events and parent-adolescent attachment among adolescents with and without mental health problems were evaluated by chi-square test (Table 1).

**Table 1:** General characteristics for the total study population at baseline and by mental health at follow-up (N = 3181).

		Mental health at follow-up		
	Total	Normal	Borderline/ Abnormal	P value (χ^2^)
	(N = 3181)	(n = 2705)	(n = 476)	
**Gender**				
Boys	51.0	52.2	44.3	0.002
**Age** (mean = 12.5, SD = 0.62)				
< 13 years	56.2	56.3	55.4	0.692
**Ethnicity**				
Dutch	48.4	49.0	44.6	0.076
**Level of education**				
Basic or theoretical pre-vocational education	50.1	48.3	60.7	<0.001
**Life events**				
Chronic or severe illness of parent	7.5	6.9	10.8	0.003
Chronic or severe illness of sibling	3.6	3.4	4.9	0.118
Mental illness of parent	2.4	1.7	5.9	<0.001
Mental illness of sibling	1.5	1.2	3.6	<0.001
Addiction to alcohol, drugs and/or gambling of parent	2.9	2.1	7.6	<0.001
Addiction to alcohol, drugs and/or gambling of sibling	1.6	1.4	2.5	0.082
Conflicts between parents	26.9	25.0	37.6	<0.001
Parental divorce	17.4	16.1	25.1	<0.001
Unwanted pregnancy	0.4	0.3	1.3	0.003
Victim of sexual abuse	1.3	0.9	3.2	<0.001
Victim of violence	4.9	3.6	12.0	<0.001
**Number of life events**				<0.001^a^
No life event	52.3	55.0	36.6	
One life event	32.0	32.2	31.2	
Multiple life events	15.7	12.9	32.1	
**Parent-adolescent attachment**				
Unfavourable	12.2	10.2	23.5	<0.001

a The three groups with different number of life events differed significantly from each other, with the no life event group displaying the least mental health problems (10.4%) and the multiple life events group showing the highest rate of mental health problems (30.3%).

Binary logistic regression analyses were conducted to assess the association between life events, parent-adolescent attachment and mental health status at follow-up (Table 2).

**Table 2:** Bivariate and multivariate associations of life events and parent-adolescent attachment with mental health problems (N = 3181).

	Bivariate^1^		Multivariate^1^	
	OR	95% CI	OR	95% CI
**Life events**				
Chronic or severe illness of parent	**1.57**	1.13 – 2.19**	1.34	0.94 – 1.90
Chronic or severe illness of sibling	1.43	0.89 – 2.29	1.23	0.75 – 2.04
Mental illness of parent	**3.37**	2.08 – 5.47***	**1.86**	**1.08 – 3.21** *
Mental illness of sibling	**2.97**	1.63 – 5.44***	1.91	0.98 – 3.73
Addiction of parent	**3.64**	2.36 – 5.63***	**2.34**	1.45 – 3.79**
Addiction of sibling	1.58	0.82 – 3.06	0.82	0.39 – 1.71
Conflicts between parents	**1.85**	1.50 – 2.27***	**1.51**	1.21 – 1.88***
Parental divorce	**1.64**	1.30 – 2.08***	1.25	0.97 – 1.62
Unwanted pregnancy	**4.22**	1.44 – 12.33**	2.17	0.63 – 7.45
Victim of sexual abuse	**3.02**	1.56 – 5.83**	1.11	0.50 – 2.50
Victim of violence	**3.66**	2.59 – 5.19***	**2.51**	1.69 – 3.70***
**Unfavourable parent-adolescent attachment**	**2.65**	2.07 – 3.40***	**2.03**	1.55 – 2.65***
Nagelkerke R^2^			0.10	

1 Bivariate and multivariate analyses included confounders: age, sex, ethnicity and education level.

* p<0.05 ** p<0.01 *** p <0.001.

Note: Bold numbers indicate significant *P*-values.

Odds ratios (OR) and their corresponding 95% confidence intervals (95% CI) were calculated. First, bivariate analyses were used to assess the association between life events and mental health status at follow-up and to assess the association between parent-adolescent attachment and mental health status at follow-up, adjusting for confounders (i.e. age, gender ethnicity and education). Second, a multivariate analysis using an enter method was performed incorporating all life events, parent-adolescent attachment and all confounders. Life events were checked for multicollinearity (all Phi correlation coefficients ≤ 0.17). Because multicollinearity was not present among the life events, all life events were entered in the same model.

To study if and to what extent parent-adolescent attachment modified the effect of one life event or multiple life events on mental health status, interaction effects were analysed on the additive scale (Table 3). Interaction on an additive scale means that the combined effect of two risk factors is different from (larger or smaller than) the *sum* of the individual effects of the factors [42]. Because we included a protective factor in our study, i.e. parent-adolescent attachment, this factor was recoded to a risk factor before calculating the interaction effect [42]. As a measure of interaction on the additive scale we present the Relative Excess Risk due to Interaction (RERI) and their 95% confidence intervals, using the delta method in Excel [43], [44]. RERI considers absolute risk and is positive (> 0) when the joint effect of risk factors is greater than the product of the effects of the individual factors. RERIs were calculated with mental health status as outcome measure at follow-up. RERIs are calculated using the following formula [42]:

**Table 3:** Interaction effect of parent-adolescent attachment and life events on mental health (N = 3181).

Life events		Parent-adolescent attachment		Mental health	OR	95% CI	RERI	95%CI
			Total	Borderline/ Abnormal				
			n	n				
One life event	Not present^1^	Favourable	1532	157	1.00		1.56	0.15 – 2.96
	Not present^1^	Unfavourable	101	13	1.29	0.70 – 2.36		
	Present	Favourable	850	105	1.23	0.95 – 1.60		
	Present	Unfavourable	143	38	3.07	2.04 – 4.63		
Multiple life events	Not present^1^	Favourable	1532	157	1.00		3.32	0.80 – 5.84
	Not present^1^	Unfavourable	101	13	1.29	0.70 – 2.37		
	Present	Favourable	360	92	2.86	2.14 – 3.84		
	Present	Unfavourable	131	57	6.47	4.39 – 9.55		

Analyses included confounders: age, sex, ethnicity and education level.

1 The reference group is no life event.

RERI = OR_A+B+_ – OR_A+B-_ – OR_A-B+_ + 1

RERI = 0 means no interaction or exact additivity; RERI > 0 means positive interaction or more than additivity; RERI < 0 means negative interaction or less than additivity; RERI can range from – infinity to + infinity.

As part of this analysis we also calculated the proportion attributable to interaction (proportion of the combined effect that is due to interaction) using the following formula [42]:

AP  =  RERI/OR_ A+B+_

AP  =  0 means no interaction or exact additivity; AP > 0 means positive interaction or more than additivity; AP < 0 means negative interaction or less than additivity; AP can range from -1 to +1.

Analyses were conducted using SPPS version 20 and Excel. Results were considered significant at *p*<0.05.

**Non-response analysis.** A comparison of adolescents included in this study (*N* = 3181) with adolescents who were excluded due to non-participation at follow-up (*N* = 5091) did not indicate significant differences in terms of gender (χ^2^ = 0.70; *p* = 0.40) and parent-adolescent attachment (χ^2^ = 1.20; *p* = 0.27). However, differences were found with regards to education, age, ethnicity and life events, with the excluded group being lower educated (χ^2^ = 151.53; *p*<0.001), older (χ^2^ = 5.94; *p*<0.05), more often of Dutch ethnicity (χ^2^ = 47.68; *p*<0.001), and with more life events (χ*^2^* = 55.22; *p*<0.001) than the adolescents who were included.

## Results

### Descriptive information

As can be seen in Table 1, the average age of adolescents in the current sample was 12.5 years (*SD*  =  0.62); 51.0% of the sample consisted of boys and 48.4% was of Dutch ethnicity. Regular conflicts between parents during the past two years was the most frequently reported life event that adolescents had experienced (26.9%). At baseline, 32.0% of the adolescents reported one life event and 15.7% reported multiple life events. Girls and lower educated adolescents had significantly more mental health problems at follow-up than boys (χ^2^ = 10.04; *p* = 0.002) and higher educated adolescents (χ^2^ = 25.03; *p*<0.001).

### Life events and mental health status

Table 1 shows the distribution of specific life events and the number of life events for the total sample, and for adolescents with normal and borderline/abnormal mental health at follow-up. The three groups with different numbers of life events differed significantly from each other (χ^2^ = 118.82; *p*<0.001), with the no life event group displaying the least mental health problems (10.4%) and the multiple life events group showing the highest rate of mental health problems (30.3%).

The presence of each specific life event, with the exception of Chronic or severe illness of sibling and Addiction of sibling, was related to a significantly increased risk of mental health problems in bivariate analyses (Table 2). After adjusting for other life events and parent-adolescent attachment, all ORs decreased and only Addiction of a parent, Mental illness of a parent, Conflicts between parents and Victim of violence were still significantly associated with mental health problems.

### Parent-adolescent attachment relationship and mental health status

An unfavourable parent-adolescent attachment at baseline was related to an increased risk of mental health problems at follow-up (see Table 2). After adjusting for the life events, the OR remained significant (OR 2.03; 95% CI 1.55 – 2.65).

### Interaction effect of parent-adolescent attachment relationship and life events on mental health

**Figure 1:**
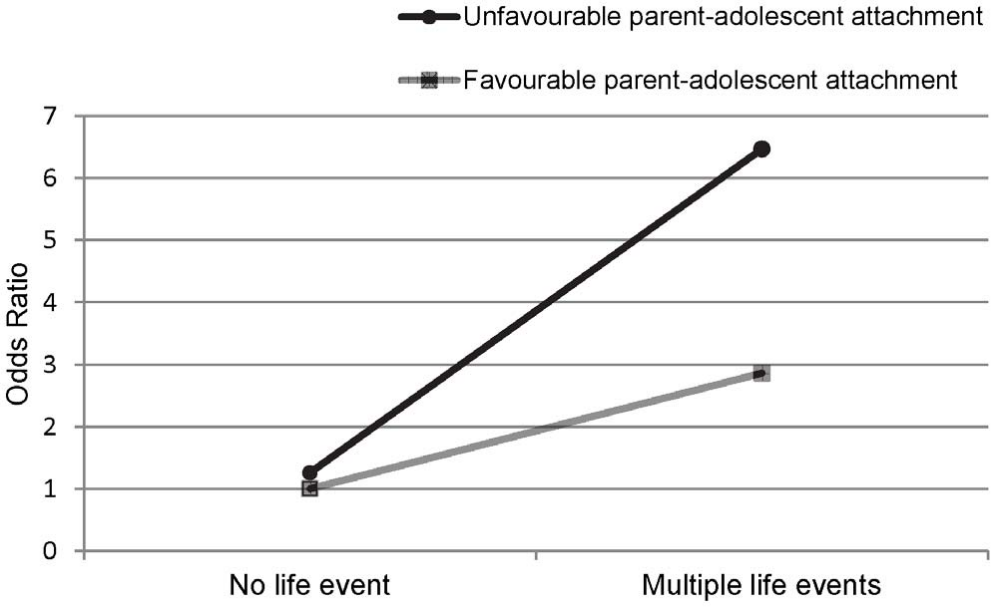
Interaction effect of parent-adolescent attachment and multiple life events on mental health.

As shown in Table 3, parent-adolescent attachment interacts with life events on mental health outcome. The combined effect of an unfavourable parent-adolescent attachment and life events on mental health was larger than the sum of the two individual effects. An unfavourable parent-adolescent attachment was associated with a higher risk of mental health problems among adolescents with one life event (RERI 1.56; 95% CI 0.15 – 2.96) and multiple life events (RERI 3.32; 95% CI 0.80 – 5.84) compared to those without a life event. Figure 1 displays the parent-adolescent attachment – multiple life events interaction effect on mental health outcome. The proportion of the combined effect that is due to interaction (AP) was 0.51 in the group with an unfavourable parent-adolescent attachment and one life event, and 0.51 in the group with an unfavourable parent-adolescent attachment and multiple life events. This indicates that 51% of the combined effect can be attributed to the interaction between parent-adolescent attachment and life events. Interaction analyses were repeated for the subgroups of age, gender, ethnicity and education; these analyses yielded similar results (data not shown, results available upon request).

## Discussion

This study shows that negative life events and parent-adolescent attachment relationship quality were associated with mental health problems in adolescents. More importantly, an interaction between the parent-adolescent attachment relationship and one and multiple life events on adolescents’ mental health was found.

This study confirms the results of earlier studies indicating a clear relationship between life events and mental health problems among adolescents [11]–[19]. A particularly high impact on mental health was observed among victims of violence. Consistent with other studies, some life events were found to no longer be significantly linked to mental health after controlling for the other life events [31], thus reflecting that some life events often co-occur. Higher rates of mental health problems were shown when multiple life events occurred together. This is also in line with previous studies, suggesting a higher probability of mental health problems when several life events accumulate [31], [45]. Furthermore, the results fit with indications by other studies that a favourable parent-adolescent attachment may be a protective factor for mental health problems in adolescents [20]–[25].

We were particularly interested in the interaction effects of parent-adolescent attachment and life events on mental health because most studies fail to examine this effect. An interaction effect of parent-adolescent attachment and life events on mental health was observed in this study. The combined effect of an unfavourable parent-adolescent attachment and life events on mental health was larger than the sum of the two individual effects, with more than half of the combined effect being due to the interaction. Thus, it seems important to not only look at direct associations, but also to assess the presence of an interaction between these factors in future research. In line with our hypothesis, these results seems to suggest that a favourable parent-adolescent attachment may serve as a buffer on the association between one or multiple life events and the mental health of adolescents. A potential explanation of the interaction found in this study could be that a favourable parent-adolescent attachment enhances adolescent’s coping abilities. Coping theory suggests that when individuals encounter potentially stressful situations one of the things they do is to evaluate their resources (e.g. parent-adolescent relationship) to handle the situation [46]. In this appraisal process, if individuals decide their internal or external resources are adequate to handle the situation, then they are not likely to feel threatened and thereby leading to more adaptive coping efforts. So, when adolescents are experiencing life events they possible could better cope with these life events if they have a favourable parent-adolescents attachment instead of an unfavourable parent-adolescent attachment.

Among adolescents who reported no life events, there was no association between the quality of the parent-adolescent attachment and their mental health status. This is in line with the findings from Wille et al. [31]. An explanation could be that individuals only benefit from protective factors, such as a favourable parent-adolescent attachment, in the presence of a risk factor [26].

There are strengths and limitations to this study that have to be mentioned. One strength of this study is that it was embedded in a longitudinal study. Also, the data set provided a unique opportunity to explore relations between particular variables of interest within a large sample. An innovative aspect of this study is that it looked not only at direct associations among the variables of interest but also at interaction effects.

However, this study also has some limitations. First, adolescents were excluded due to

non-participation at follow-up. In a non-response analysis we showed that the excluded group was lower educated, older and more often of Dutch ethnicity. Although we included these

variables as confounders in our analyses, the current findings should be generalized with caution, and we propose replication in large and varied populations. Second, as with any self-report survey, adolescents’ self-report could be biased. Although it would have been preferably to use multiple informants, research suggests that adolescents are better reporters of their own mental health status than parents and teacher. For example, adolescents’ self-reported mental health status corresponded better with independent psychiatric assessment than parent or teacher [47]. Nevertheless, results of this study have to be interpreted with caution and we recommend future studies to use multiple informants. Third, a total life event score was calculated, which makes it not possible to distinguish, for example, the interaction between parent-adolescent attachment and life events that are (at least partly) related to the adolescents’ own behaviour (behaviour-dependent events), such as conflicts with parents or peers, and those that are independent of their behaviour (behaviour-independent), such as natural disasters. Therefore we cannot distinguish if a favourable parent-adolescent attachment may be particularly beneficial for adolescents exposed to particular life events, and/or not beneficial for adolescents exposed to other life events.

Furthermore, as our main aim was to predict the *occurrence* of mental health problems at follow-up. Therefore, we choose not to adjust for baseline mental health in the analyses because this would have only allow us to draw conclusions about the influences of an unfavourable parent-adolescent attachment and life events on *changes* in mental health between follow-up and baseline. In that case, we would not have taken into account the impact that an unfavourable parent-adolescent attachment and life events already had on the mental health of adolescents at baseline. The unfavourable parent-adolescent attachment and life events could have been present earlier than at baseline. So, due to the nature of our research question (i.e. about the occurrence of mental health), and not changes in mental health (i.e. incidence), we did not adjust for baseline mental health in this study. Thereby, we studied whether there is a long term relationship and interaction effect between parent-adolescent attachment, life events and mental health. However, it must be noted that causality cannot be inferred from these analyses, because it is unknown for example whether mental health problems were already present when life events occurred or wether life events, parent-adolescent attachment and mental health problems have mutually influenced each other.

To disentangle the questions posed above, future research with a prospective design that enables researchers to examine the temporal ordering of the variables is needed. However, this is difficult, because life events and mental health issues are often already present at very young age. Long-term longitudinal cohort studies that follow children from fetal life onwards are therefore desirable. Furthermore, better understanding of why the parent-adolescent attachment could serve as a buffer on the relation between life events and mental health problems is necessary. As we mentioned before, it is possible, for example, that a favourable parent-adolescent attachment enhance adolescent’s coping abilities, which mediates the relation between life events and mental health problems. Therefore, future studies should integrate moderator and mediator research by testing for specific mediators (e.g. threat or coping abilities) in relation to parent-adolescent attachment as a moderator, so that we can further test our hypotheses and better understand the complex way in which life events affect the mental health of adolescents. Further, experimental studies in which the parental-adolescent attachment will be enhanced by an intervention can give more information about the causal influence the parent-adolescent attachment relationship may have on mental health problems.

In conclusion, results from the current study support earlier studies indicating an association of negative life events and parent-adolescent attachment with mental health problems. Results also support an interaction effect between parent-adolescent attachment and negative life events on mental health. This seems to support the hypothesis that a favourable parent-adolescent attachment relationship may serve as a buffer for adolescents with one or multiple life events. However, conclusions about causality cannot be drawn from this study and examining the effects of life events and an unfavourable parent-adolescent attachment on adolescents’ mental health simplifies the complex processes in the development of mental health problems in which a large number of factors play a role. Nonetheless, it enables the identification of adolescents with a high probability of displaying disturbed development. Adolescents with one or multiple life events and an unfavourable parent-adolescent attachment seems to be a vulnerable group for mental health problems. Future research needs to continue to probe the reasons why some adolescents with life events are functioning better than others. This knowledge would be helpful in designing effective prevention and intervention programs for adolescents exposed to life events.
